# The urothelium of a hibernator: the American black bear

**DOI:** 10.14814/phy2.12429

**Published:** 2015-06-24

**Authors:** David A Spector, Jie Deng, Richard Coleman, James B Wade

**Affiliations:** 1 Division of Renal Medicine, Johns Hopkins Bayview Medical Center, The Johns Hopkins University School of MedicineBaltimore, Maryland, USA; 2 Department of Physiology, University of Maryland School of MedicineBaltimore, Maryland, USA

**Keywords:** Black bear, hibernation, urinary bladder microscopy, urine reabsorption, urothelium

## Abstract

The American black bear undergoes a 3–5 month winter hibernation during which time bears do not eat, drink, defecate, or urinate. During hibernation renal function (GFR) is 16–50% of normal but urine is reabsorbed across the urinary bladder (UB) urothelium thus enabling metabolic recycling of all urinary constituents. To elucidate the mechanism(s) whereby urine is reabsorbed, we examined the UBs of five nonhibernating wild bears using light, electron (EM), and confocal immunofluorescent (IF) microscopy–concentrating on two components of the urothelial permeability barrier – the umbrella cell apical membranes and tight junctions (TJ). Bear UB has the same tissue layers (serosa, muscularis, lamina propria, urothelia) and its urothelia has the same cell layers (basal, intermediate, umbrella cells) as other mammalians. By EM, the bear apical membrane demonstrated a typical mammalian scalloped appearance with hinge and plaque regions – the latter containing an asymmetric trilaminar membrane and, on IF, uroplakins Ia, IIIa, and IIIb. The umbrella cell TJs appeared similar to those in other mammals and also contained TJ proteins occludin and claudin - 4, and not claudin –2. Thus, we were unable to demonstrate urothelial apical membrane or TJ differences between active black bears and other mammals. Expression and localization of UT-B, AQP-1 and -3, and Na^+^, K^+^-ATPase on bear urothelial membranes was similar to that of other mammals. Similar studies of urothelia of hibernating bears, including evaluation of the apical membrane lipid bilayer and GAGs layer are warranted to elucidate the mechanism(s) whereby hibernating bears reabsorb their daily urine output and thus ensure successful hibernation.

## Introduction

A number of bear species, including the American black bear (*Ursus americanus*), undergo a winter hibernation for 4–5 months during which time bears do not eat, drink, defecate, or urinate (the latter documented in: Nelson 1973 [includes earlier references], Toien et al. 2011; Folk et al. 1972; Nelson et al. 1973; Barboza et al. 1997). Remarkably, despite lack of urination, hibernating bears do not become uremic and blood urea, although not creatinine, concentrations actually fall during hibernation (Nelson et al. 1973, 1984; Nelson 1978; Hellgren et al. 1990; Barboza et al. 1997; Tajana and Cervigni 2013). The mechanisms, whereby hibernating bears accomplish this metabolic feat are unknown. Other mammals (including active/summer bears (Nelson et al. 1975) similarly restricted food and water or made anuric become uremic or die – making the hibernating bear a unique animal model for studying many aspects of renal failure and bladder function (Nelson 1973; Singer 2002; Stenvinkel et al. 2012; Jani et al. 2013).

Despite the absence of urination, there is evidence that bear kidneys are making urine during hibernation. Thus, the urinary bladder catheterization in hibernating bears has yielded 24 h urine volumes and random catheterization volumes (also reflecting 24 h urine volume, see below) in the range of 70–180 mL (Brown et al. 1971; Nelson et al. 1973, 1975; Folk et al. 1974) while standard inulin and creatinine clearance studies during hibernation revealed glomerular filtration rates (GFR) of 12–64 mL/min – representing 16–50% of summer GFR in the same bears (Brown et al. 1971). The small volumes of the reported daily urine during hibernation are due in part to the small size of many of the bears utilized for the studies, to the relatively highly concentrated urine (hibernation mean = 1116 mOsm/kg, nonhibernation mean = 227 mOsm/kg, (Brown et al. 1971), and to the reduced urea and other solute load (consequent to lack of food intake) requiring excretion – as documented by solute excretions in 24 h urine collections by Nelson (1973) and Nelson et al. (1973) – which would result in predictably smaller urine outputs. Since hibernating bears do not urinate despite documented continued urine production, Nelson concluded that their daily urine output must be daily matched by (roughly) identical volumes of urine reabsorption across the urinary bladder (Nelson et al. 1973, 1975). Thus, hibernating bear bladders must remain partially filled and randomly catheterized bladder urine volumes must approximate 24 h urine volumes. In support of the notion of reabsorption of urinary constituents across the bladder wall, Nelson instilled [14C] urea and D_2_O into the bladder urine of hibernating bears and reported rapid reabsorption of both isotopes across bladder urothelia and their simultaneous appearance in plasma – thus documenting unexpected bladder reabsorption of water and urea (Nelson et al. 1975). Nelson concluded that bear urinary bladder plays a central role in the conservation and recycling of water and nitrogenous wastes, (and, presumably, all other urinary constituents) during hibernation. Since mammalian bladder has long been felt to act as an impermeable short-term transit and storage vehicle for urine made by the kidneys (Hicks 1975) and to be relatively impermeable to urine constituents (Chang et al. 1994; Negrete et al. 1996; Zeidel 1998), Nelson's conclusions were unexpected and the mechanism(s) whereby bladder urine might be reabsorbed in hibernating bears remains unknown. Recent data, however, demonstrates that mammalian urothelia (the epithelial cell lining of the urinary tract from renal pelvis to the proximal urethrea) in nonhibernators is a surprisingly dynamic and complex tissue with investigators demonstrating umbrella cell release of numerous active substances including: proteases, hormones, and mediators such as ATP, adenosine, and acetylcholine, as well as cell surface ion channels and receptors for multiple mediators, growth factors, and hormones (Khandelwal et al. 2009; Birder and Andersson 2013). Furthermore, a number of in vivo studies in several mammalian species have demonstrated regulated water, nitrogenous wastes and solute transport across urothelium (Levinsky and Berliner 1959; Holbrugger 1987; Walser et al. 1988; Spector et al. 2007, 2011, 2012, 2013). Thus, at least small quantities of vectorial solute and water transport across urinary tract urothelia may be a feature of most or all mammalian species and the hibernating bear may or may not have unique urothelial transport capabilities enabling the net transport of urinary constituents.

The urothelial permeability barriers in other mammalian species are thought to reside in the apical membrane (including both the unique lipid bilayer and the associated paracrystalline array of transmembrane uroplakin proteins) of the large “umbrella” cells lining the urinary tract lumens, the tight junctions between those cells, and likely in the adherent urinary glycosaminoglycans (GAGs) layer overlying the apical membrane (Hurst et al. 1987; Parsons et al. 1990; Calderon et al. 1998; Zeidel 1998; Lewis 2000; Hu et al. 2002; Khandelwal et al. 2009; Tajana and Cervigni 2013). The apical membranes of these cells have been shown in vitro to have very low permeability to water, urea, and ammonia albeit with some species differences (Chang et al. 1994; Negrete et al. 1996; Zeidel 1996, 1998). Since there have been no anatomic or functional studies of bear urinary bladder, it is unknown if the same urothelial permeability barriers are present in bears and/or if they are altered during hibernation. Regardless, there are a number of physiologic factors that might be expected to promote reabsorption of water and solutes across these barriers, including the large urinary tract luminal surface area, long storage time, large concentration gradients for solutes, and ions, and potential mechanical and pressure stimuli. In the case of hibernating bears, the apparent continuous presence of urine in the bladder may greatly magnify the effect of some of these factors and may result in unique functional properties. Furthermore, water and solute channels and transporters known to facilitate and regulate water and solute transport across renal epithelia have recently been discovered in urothelial cells in many mammalian species. These include the aquaporin water channels (Spector et al. 2002; Rubenwolf et al. 2009), urea transporters (Spector et al. 2004, 2007; Lucien et al. 2005; Walpole et al. 2014) and transporters for sodium (Smith et al. 1998; Lewis 2000; Wang et al. 2003), chloride (Wang et al. 2003), and potassium (Spector et al. 2008; Sun et al. 2007; Wang et al. 2003; reviewed in Khandelwal et al. 2009). Whether these and other channels and transporters are present in bear species and whether they might be up-regulated to participate in increased reabsorption phenomenon during hibernation is unknown.

In spite of the apparent importance of the bladder to successful hibernation in bears, and the rapidly increasing discoveries regarding the complexities of mammalian urothelial functioning, there has been no previous microscopic or functional description of bear bladder. Whether the black bear has unique urinary bladder architecture, urothelial solute and/or water channels and transporters, or permeability barriers, is unknown. Here we describe the results of light, electron, and immunofluorescent microscopy of the urothelial layer of urinary bladders in five wild black bears, with an emphasis on those components thought to make up the primary urothelial permeability barriers – the umbrella cell apical (lumenal) membrane (and associated subapical cytoplasmic vesicles) and the tight junctions between umbrella cells – as well as representative urothelial cell channels and transporters, and compare these results with similar studies in rats.

## Methods

All research reported herein adheres to the “Guiding Principles in The Care and Use of Animals” of the American Physiological Society and was approved by the Johns Hopkins University Animal Care and Use Committee.

### Bears

All bear tissues used in these experiments were obtained from free-roaming wild Black bears living in the States of Maryland or Pennsylvania. Bears were euthanized by State natural resource officials either because of bear cub abandonment or for purposes of protecting humans, animals, or property utilizing carbon monoxide or gunshot as per State policy. Characteristics of the five bears whose tissues were examined for purposes of this study are listed in Table1.

**Table 1:** Characteristics of wild black bears

Bear	Age/Weight/Sex	Date euthanized	State	Comments
1	3 month/F	4/7	Pennsylvania	Abandoned cub
2	3–4 months/N/A	4/23	Pennsylvania	Separated from sow
3	9 months/48 lb/F	10/17	Maryland	Struck by car; euthanized
4	2 years/105 lb/F	5/27	Maryland	Nuisance bear; chased by dogs
5	212 lb/M	1/22	Pennsylvania	Nuisance bear; denned under habitated house; diseased: Mange

All bears were thought to be healthy except for bear #5 which suffered from mange and appeared thin, and bear #3 which had just been struck by a car when found. Bears #1–4 were not hibernating; bear #5 was winter denning under a porch in a habitated house, but not hibernating as it was noted to be active and foraging. Bear #4 was captured after a prolonged chase by dogs and humans. All five bears were considered to be under severe “stress” due to circumstances prior to and/or related to their capture for hours to days prior to euthanasia. All bladders were removed from bears within 60–120 min and postmortem by State officials (in Pennsylvania by Walter O. Cottrell, wildlife veterinarian, Pennsylvania Game Commission, and in Maryland by Harry Spiker, Director Bear Project, Department of Natural Resources), and tissue samples (bladders were cut into two halves) were immediately placed in 10% buffered formaldehyde or chilled 4% glutaraldehyde for fixation. Care was taken not to disturb the epithelial cells lining the bladders as far as possible. Tissues were stored locally for two to 20 days, and then delivered overnight to our laboratory where they were refrigerated and stored, and recut for various microscopic techniques. Tissues designated for immunocytochemistry were usually rinsed in buffered phosphate solution prior to embedding, and all formaldehyde-fixed tissues were embedded in paraffin blocks, using standard techniques in preparation for light and immunofluorescent microscopy.

### Rats

Female Sprague–Dawley rats (Harlan, Indianapolis, IN) weighing 200–240 g were maintained on ad libitum intake of chow (#2018, Harlan Teklad) and water. Most rats underwent 48 h water-restriction or water loading as previously described (Spector et al. 2011, 2013). Rat bladders were removed from anesthetized rats and fixed in paraformaldehyde in PBS for immunolocalization and light microscopy, or fixed in 3 or 4% glutaraldehyde in 0.1 m sodium phosphate buffer (pH 7.3) for transmission electron microscopy.

### Light microscopy

Tissues were embedded in paraffin blocks and sectioned at 3–5 *μ*m; sections were affixed to glass slides and stained with hematoxylin and eosin by The Johns Hopkins Hospital Reference Histology Laboratory.

### Immunofluorescence microscopy

Bladder cross-sections were cut at 3 *μ*m thickness from paraffin blocks and picked up on chrome-alum gelatin-coated glass coverslips and dried on a warming plate. The sections were then deparaffinized in two xylene baths and two absolute ethanol baths, 5 min each, and rehydrated in a graded ethanol series to distilled water. For epitope retrieval, the coverslips were placed in a pH 8 aqueous solution of Tris (1 mmol/L), EDTA (0.5 mmol/L) and SDS (0.02%). The retrieval solution was heated to boiling in a microwave oven, transferred to a conventional boiling water bath for 15 min and then allowed to cool to room temperature before the sections were thoroughly washed in distilled water to remove the SDS. Sections were preincubated for 30 min with Image-iT blocking solution (Invitrogen, now Life Technologies, Carlsbad, CA), rinsed in PBS, then preincubated an additional 30 min in a solution of 2% BSA, 0.2% fish gelatin, 5% normal donkey serum, and 0.2% sodium azide in PBS. Tissues were thoroughly rinsed with Tris-buffered saline (TBS) to remove PBS. Incubations with specific antibiodies (as described above), diluted in TBS containing 1% BSA, 0.2% fish gelatin, 0.1% Tween 20, 10 mmol/L CaCl_2_ and 0.2% sodium azide, took place overnight in a humid chamber at 4°C. After thorough washing in high- salt wash (incubation medium plus added sodium chloride at 0.5M), two or three antibodies were localized on the same section detected with Alexa Fluor 488 and 568-conjugated donkey antibodies (Jackson ImmunoResearch Laboratories, Inc., West Grove, PA), and DyLight 649-conjugated donkey antibody (BioLegend, San Diego, CA). Labeled tissues were examined using standard immunofluorescent confocal microscopy.

### Antibodies

Antibodies to AQP-1 and -3 were raised in both rabbit and chicken. These antibodies have been extensively characterized and previously used in urinary tract tissue by the authors (Spector et al. 2002). Antibodies to UTB raised in rabbits were generously supplied and have been extensively characterized by Drs. Janet Klein and Jeff M. Sands (Atlanta, GA) and previously used in urinary tract tissue by the authors, (Spector et al. 2004, 2007). Antibodies to uroplakins were generously supplied by Dr. Tung-Tien Sun, (New York University School of Medicine, NY, NY). These previously described antibodies all raised in rabbits included: “Total” Bovine Uroplakins prepared against the total uroplakin proteins of bovine urothelial plaques (NYC 745: Yu et al. 1990; Wu et al. 1990), Uroplakin IIIa (UPIIIa, R182: Liang et al. 2001), Uroplakin IIIb (UPIIIb, c-6177: Deng et al. 2002), Uroplakin Ia (UP Ia, R-4867: Liang et al. 2001), Antibody to Sodium Potassium ATPase (an alpha 1 monoclonal mouse antibody) is from Upstate (now Millipore). Antibodies raised in mice to Claudins -2 and -4 were obtained from Zymed (now Life Technologies, Grand Island, NY), and those to occludin raised in mice were obtained from Transduction Labs. Both primary and secondary antibodies were used at a concentration of 10 *μ*g/mL by dilution of PBS containing 1% BSA, 0.2% fish gelatin, 0.1% Tween 20, and 0.2% sodium azide.

### Transmission electron microscopy

Transmission electron microscopy was performed by The Johns Hopkins Hospital Pathology Department electron microscope facility. Tissues fixed in 4°C glutaraldehyde were rinsed in 0.1 m sodium phosphate buffer, postfixed in 1% osmium tetroxide in the same buffer for 1 h at room temperature. The tissues were dehydrated in a graded series of ethanol, transitioned with toluene, followed by infiltration, and embedding in epoxy resin EPON 812 (Polysciences, Inc., Warrington, PA). Semi-thin sections of 500 nm thickness were cut and stained with 1% toluidine blue for visualization by light microscopy. Ultra-thin pale gold sections of selected areas were cut at a thickness of approximately 100 nm with a diamond knife (DIATOME), placed on 200 mesh copper grids, and dried at 60°C for 10 min. Sections were stained with a saturated solution of uranyl acetate for 10 min followed by lead citrate for 2 min. Sections were examined with a transmission electron microscope (Philips CM 12 TEM; Philips, Eindhoven, the Netherlands), using a tungsten filament operating at an accelerating voltage of 60 keV. Images were acquired using a Morada 11 Megapixel side-mounted TEM CCD camera (Olympus Soft Imaging Solutions, now EMSIS Gmbh, Munster, Germany).

## Results

### Bladder wall architecture

**Figure 1:**
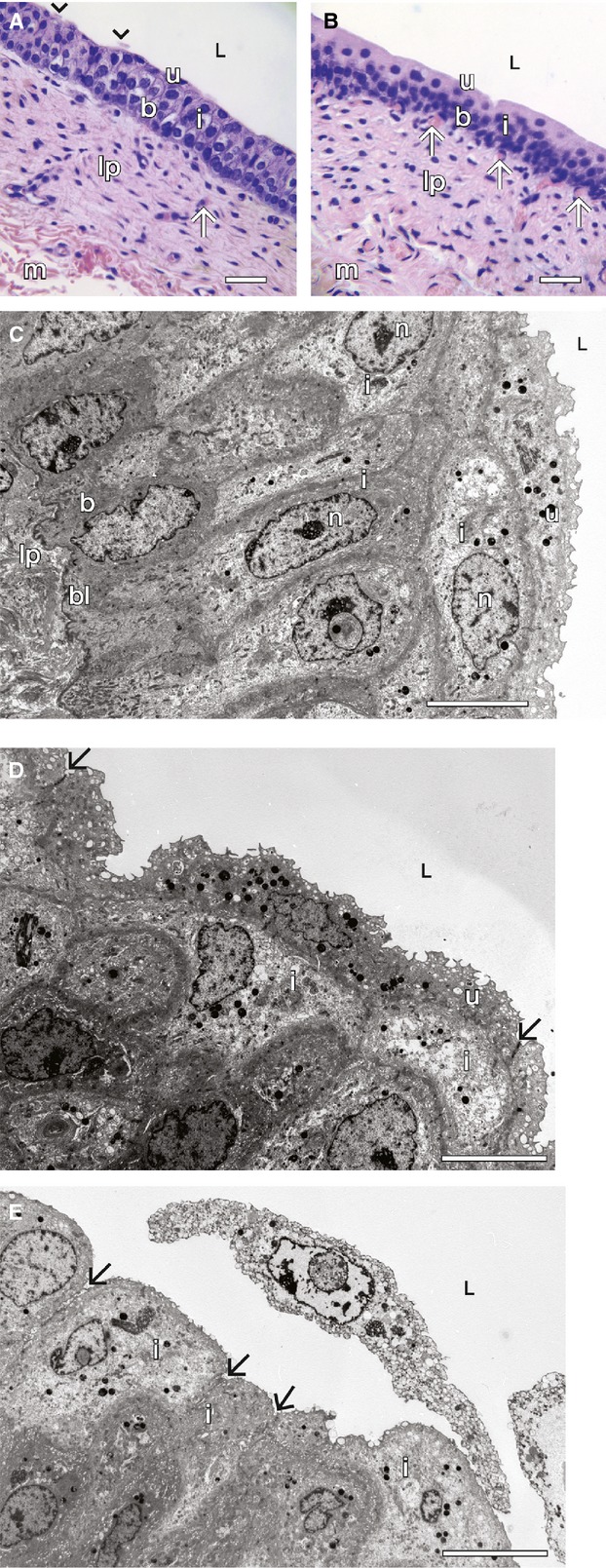
(A) Bear bladder. Light microscopy, H&E stain, showing muscularis (m), lamina propria (lp), and urothelial layer – consisting of basal cells (b), intermediate cells (i), and umbrella cells (u); L, lumen. Arrows indicate subepithelial capillaries. Arrowheads denote umbrella cell disruption/desquamation. Scale bar = 30 *μ*m. (B) Rat bladder. Light microscopy, H&E stain, showing muscularis, lamina propria, and urothelial layer. Symbols and scale bar identical to (A). (C) Electron microscopy of bear urothelium, showing 4–5 cell layers. Symbols identical to (A). bl, basal lamina; n, nucleus. Scale bar = 10 *μ*m. (D) Electron microscopy of bear urothelium demonstrating wide, thin umbrella cell overlaying intermediate cells, symbolic identical to those in (A). Arrows = tight junctions, scale bar = 10 *μ*m. (E) Electron microscopy of bear urothelium showing desquamating umbrella cells with underlying intermediate cells (i) possibly undergoing phenotypic transformation to surface umbrella cells. Cytoplasmic vesicles are shown in various stages of exocytic incorporation into apical membranes and early or attenuated junctional complexes (arrows) at apical-lateral sites of adjoining transforming intermediate cells. Scale bar = 10 *μ*m.

By light and electron microscopy, the wall of the bear urinary bladder (and ureter data not shown), as in rat and other mammalian species (Hicks 1975) comprised four layers: an outermost thin serosa, a thick smooth muscle compartment, a sub-epithelial lamina propria of loose connective tissue (containing nerve fibers, myofibroblasts, blood vessels, and capillaries), and an epithelial cell layer (“urothelia”) lining the bladder lumen (Fig.1A) and separated from the subepithelial blood capillaries and lamina propria by a thin basal lamina seen on electron microscopy (Fig.1C). As in the rat (Fig.1B) and most, but not all, other mammals (Firth and Hicks 1973; Khandelwal et al. 2009) the bear bladder urothelium is three to five cell layers deep and exhibits an orderly pattern of differentiation from a one-cell layer of relatively small undifferentiated basal cells, through a one to three pear (variably)-shaped intermediate cell layer, to a single-cell layer of wider highly differentiated “umbrella” cells lining the bladder lumen (Fig.1A and C).

By light and electron microscopy (Fig.1A and C), the umbrella cell layers of bears generally seems to be flatter and wider than in rats (Fig.1B) with nucleii – sometimes flattened – located closer to the luminal surface (Fig.1D). A similar appearance is present also on light microscopy of bear ureteral urothelia (data not shown). Such findings in umbrella cells in other species are characteristic of the urothelia subjected to hydrostatic pressure (Truschel et al. 2002) or distension (Hicks 1975; Minsky and Chlapowski 1978). By light microscopy there was (occasional) evidence of disruption of the umbrella cell apical surface in bears (Fig.1A), but none in rats (Fig.1B). By electron microscopy, evidence of some degree of cell damage – especially in umbrella cells, but sometimes in intermediate cells – was often present, usually in patchy distribution. These changes were more prominent in the two older bears studied and included areas of reduced cytoplasmic density, loss of subcellular structures and organization, intracellular vacuolization, and, in some instances, dilatation of the intercellular basolateral spaces with reduced cell-to-cell contact and rarely, umbrella cell sloughing (Fig.1E). Under electron and confocal immunofluorescent microscopy (see below) in the two older bears (#4, 5), typical umbrella cells were apparently absent in some areas, with exposure of underlying intermediate cells. Umbrella cells are thought to be derived, in part at least, from the layer of intermediate cells – following damage, apoptosis, or necrosis, and sloughing off of preexisting umbrella cells followed by phenotypic change in intermediate cells to typical umbrella cells (Hicks 1975; Lavelle et al. 2002; Apodaca et al. 2003; Khandelwal et al. 2009). In some areas, the bear luminal cells appeared more like intermediate cells in size and shape (Fig.1E) and may account for at least some of the apparent heterogeneity of the luminal cells and their apical membranes seen in light, immunofluorescent (see below) and electron microscopy of the bear bladder, compared to the homogeneous pattern of umbrella cells in rats (Fig.1B) and most other reported mammals (Firth and Hicks 1973; Hicks 1975).

### Apical membrane and associated subepithelial vesicles

**Figure 2:**
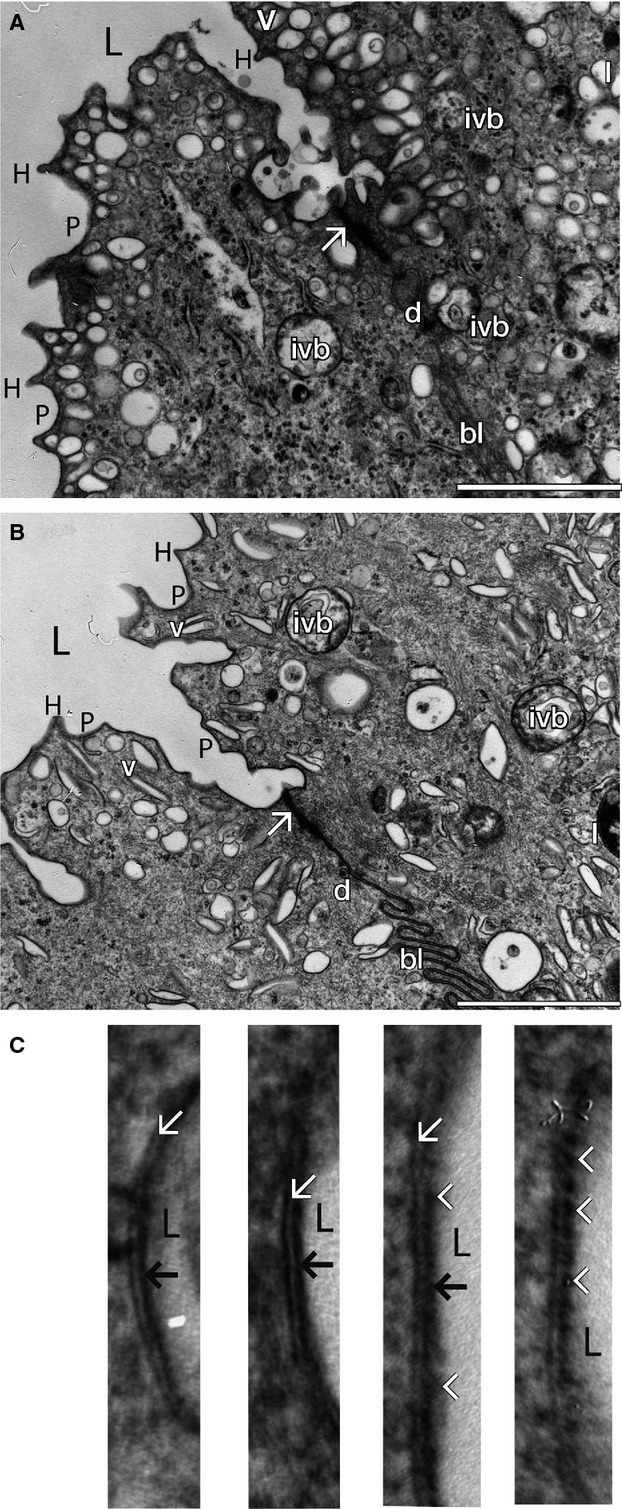
(A) Electron microscopy of bear umbrella cell apical membrane, subapical cytoplasmic vesicles, and junctional complex (arrow) comprised of the tight junction, adherens junctions, and desmosomes (d) between two adjacent umbrella cells. The apical membranes are made up of “hinge” (H) and “plaque” (P) regions. The underlying cytoplasm contains numerous discoid or oval-shaped vesicles (v), as well as larger vesicles containing inclusion bodies (ivb), and lysozymes (l). bl, basolateral membrane, scale bar = 2 *μ*m. (B) Electron microscopy of rat umbrella cell apical membrane, cytoplasmic vesicles, and junctional complex, at similar magnification as in bear (A). Many of the subapical cytoplasmic vesicles are more fusiform in shape than seen in analogous bear cells. Symbols identical to those in (A). Scale bar = 2 *μ*m. (C) Very high magnification electron microscopy of representative bear umbrella cell apical membrane (plaque regions), and sub-apical cytoplasmic vesicle (far left panel), taken from two bears. The trilaminar anatomy (a light center [open arrows] sandwiched between two dark leaflets) is apparent. In most (but not all) images the leaflet thickness/density is asymmetric with the lumenal (exofacial) leaf thicker or denser (filled arrowheads). Dense regularly-spaced membrane projections (substructure) from the exofacial leaf into the lumenal space are sometimes apparent (arrowheads – two images on right). L, lumen.

By electron microscopy, the apical membrane of bear umbrella cells demonstrates an irregular scalloped appearance with angular rigid-appearing luminal projections (“hinge areas”) and intervening concavities (“plaque” regions) comprised of a dense trilaminar (see below) membrane (low magnification: Fig.1C and D; high magnification: 2A) all of which features are almost identical to these of rat apical membrane (Fig.2B) and to those reported in other mammalian species (Koss 1969; Firth and Hicks 1973; Hicks 1975). (These findings are not present in intermediate cells except following damage to, or loss of, the overlying umbrella cells and consequent phenotypic change in the underlying intermediate cells). The apical membrane studied under very high EM magnification in bears appears to be trilaminar and similar to that reported in other species (Koss 1969; Hicks 1975; Apodaca 2004), and often (but not always, 26) demonstrates an asymmetrical appearance of the two leaflets (Fig.2C). This configuration has been termed an “asymmetric unit membrane” (Koss 1969; Hicks et al. 1974; Apodaca 2004). In other species, the electron-dense layers are comprises a crystalline array of uroplakins (Firth and Hicks 1973; Hicks et al. 1974; reviewed in Wu et al. 2009) and the central clear layer, presumably, in large part lipids. Uroplakins and lipids play independent and additive roles in preventing permeation as shown by studies of lipid membranes in using chambers (Hill and Zeidel 2000, 2003; Lande et al. 1995; reviewed in Zeidel 1998) and in uroplakin knockout mice in which (partial) barrier function persisted in spite of successful uroplakin IIIa knockout (Hu et al. 2000, 2002). Below the apical membrane in both bears and rats are (variably) numerous cytoplasmic vesicles, which were usually ovoid or discoid shaped in bears (Fig.2A), and ovoid, or fusiform shaped in rats (Fig.2B). It is possible that the preponderance of discord-shaped vesicles in bears reflects bladder stretch and the presence of mostly endocytosed vesicles (Minsky and Chlapowski 1978). As in other species, the vesicular membranes of bears are trilaminar (Fig.2C; image on far left) and seem similar or identical to apical membranes in appearance being, presumably, comprising of lipid and uroplakin constituents identical to those of the apical cell membrane (Hicks 1975; Khandelwal et al. 2009). These vesicles insert into the overlying apical membrane by exocytosis and are retrieved out of the overlying apical membrane by endocytosis during bladder contraction and expansion (Truschel et al. 2002; Minsky and Chlapowski 1978; reviewed in Khandelwal et al. 2009). In bears, as in other species (Apodaca 2004; Khandelwal et al. 2009), these vesicles are also seen in the outermost intermediate cells where they often assume a cytoplasmic location near the plasma membrane underlying and nearest the umbrella cells, and where they are often fusiform in shape as in other species (EM data not shown). Larger round vesicles-containing lamellar and vesicular inclusion bodies as well as lysosomes are also present in both bear (Fig.2A) and rat (Fig.2B) umbrella cells as is characteristic of mammalian urothelia (Koss 1969; Firth and Hicks 1973; Hicks 1975).

**Figure 3:**
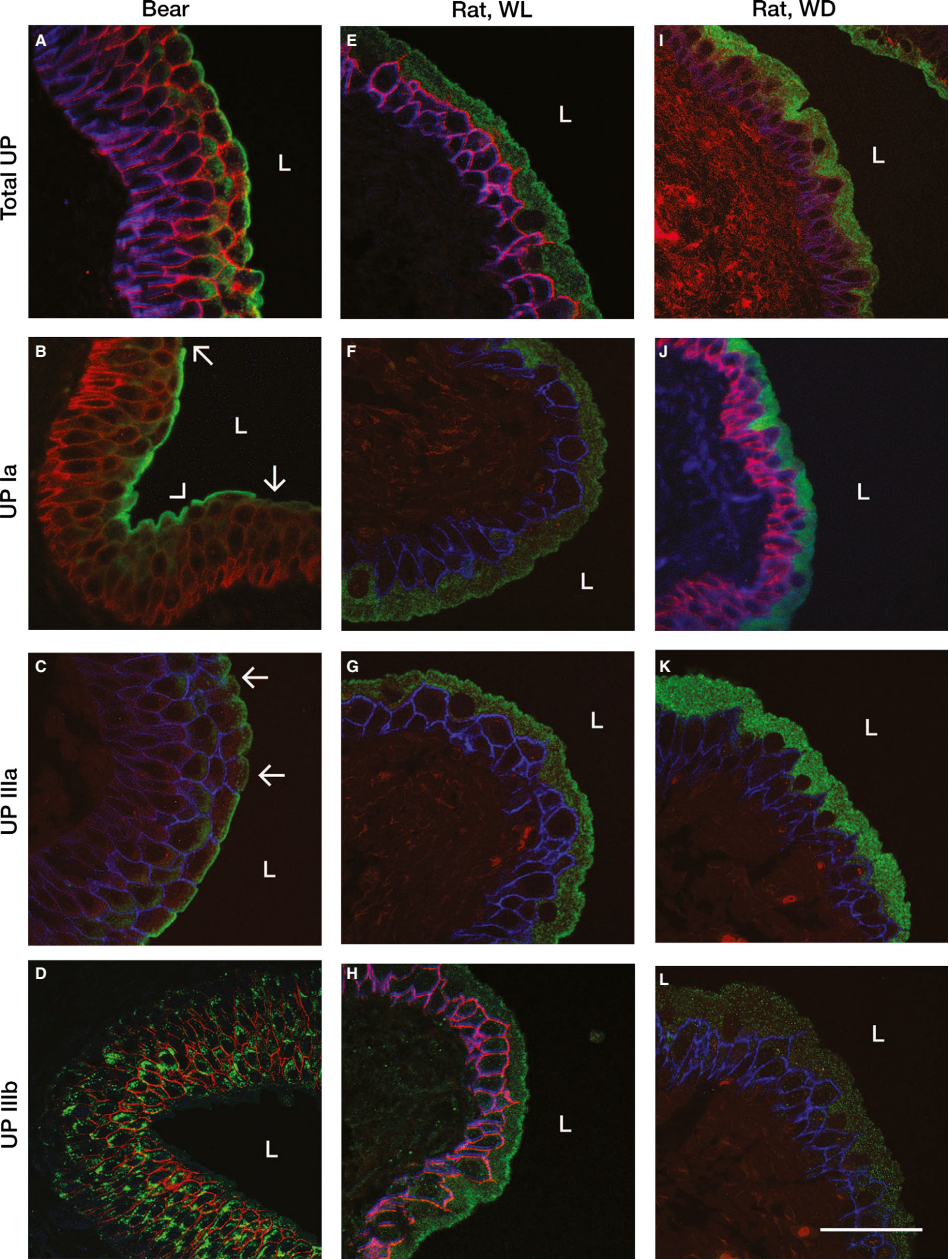
Immunofluorescent confocal microscopy of bear urothelium (left column), water-loaded rat (Rat, WL, middle column) and water-deprived rat (Rat, WD, right column), using antibodies to “total” (all) uroplakin species (Total UP, row 1), uroplakin Ia (UP Ia, row 2), uroplakin IIIa (UP IIIa, row 3), and uroplakin IIIb (UP IIIb, row 4). All uroplakin antibodies stain green. Counterstains representing claudin-4 and/or aquaporin-3, stain blue and/or red. Total uroplakins (Panel A) and uroplakins Ia (Panel B) and IIIa (Panel C) are strongly expressed in bear umbrella cell apical membrane, and less so in umbrella and intermediate cell cytoplasm. Uroplakin IIIb (Panel D) is expressed as cytoplasmic granules in bear intermediate and umbrella cells. Note gaps in Uroplakin Ia staining in bear due to loss of umbrella cell layer (arrows, Panel B), or apical membrane rupture (arrowhead, Panel B). In panel C note differences in bear urothelial lumenal cell shape and UP IIIa staining character in uppermost five umbrella cells (arrows) compared to lower five umbrella cells – possibly related to phenotypic change of cells from intermediate to umbrella cells after damage and loss of previous umbrella cells. In rats “total” and individual uroplakins are strongly expressed in both water-loaded and water-deprived conditions. However, in water-loaded rats there is mild to moderate cytoplasmic, but strong apical membrane expression of uroplakins – in contrast to water-deprived rats which have moderate to strong diffuse cytoplasmic expression without apical membrane accentuation. Scale bar = 50 *μ*m.

To determine if bear umbrella cell apical membranes are comprised of the same barrier protein uroplakins as have been described in other species (Wu et al. 2009), we carried out immunofluorescent confocal microscopy of bear urothelia utilizing antibodies to “total” (see “Methods”) bovine uroplakins, as well as to representative individual uroplakins Ia, IIIa (which in other species form heterodimers with uroplakins II and Ib [not evaluated here], respectively [95]), and IIIb. Bear urothelial cells strongly expressed “total” uroplakins in the umbrella cells apical membrane and subapical cytoplasm (presumably in vesicles) and to a lesser extent in the cytoplasm underlining the uppermost plasma membranes of the outermost intermediate cells (Fig.3A, green). Individual uroplakins Ia and IIIa were strongly expressed in bear umbrella cell apical membranes and to a lesser extent in umbrella cell and outermost intermediate cell cytoplasm (Fig.3B and C) in locations identical to those for “total” uroplakins. Uroplakin IIIb was expressed in the punctate/granular form in cytoplasm of intermediate cells but less so in bear umbrella cells (Fig.3D). Gaps, alteration, or attenuation of uroplakin (total, Ia, IIIa) expression were present in areas of all bear tissues examined – notably at sites where the apical membrane or the umbrella cell layer seemed to be altered, damaged, or lost (arrows, Fig.3B and C). Some luminal cells showed a more rounded shape and reduced uroplakin expression compared to adjacent cells (for UP IIIa, arrows, uppermost cells, Fig.3C) – consistent with cells undergoing a phenotypic change from intermediate to umbrella cells.

In comparison to rats, the same anti-uroplakins antibodies were utilized in rats subjected to conditions of water loading or water deprivation (since we have previously reported significant differences in rat urothelial solute permeability between these physiologic conditions (Spector et al. 2013, 2011). As in bears, uroplakins (“total”, Ia and IIIa) were strongly expressed in rat umbrella cells, and much less so in intermediate cells. In rats, subjected to water-loading conditions, there was a strong apical membrane localization of “total” and all individual uroplakins (Fig.3E–H). In contrast, in rats subjected to water deprivation, “total” and individual uroplakins expression were more homogeneously expressed in umbrella cell cytoplasm without noticeable apical membrane accentuation (Fig.3I–L). Localization of “total” and individual uroplakins Ia and IIIa in bear urothelia were generally more like that of rats undergoing water loading, rather than water deprivation.

### Junctional complexes of umbrella cells

**Figure 4:**
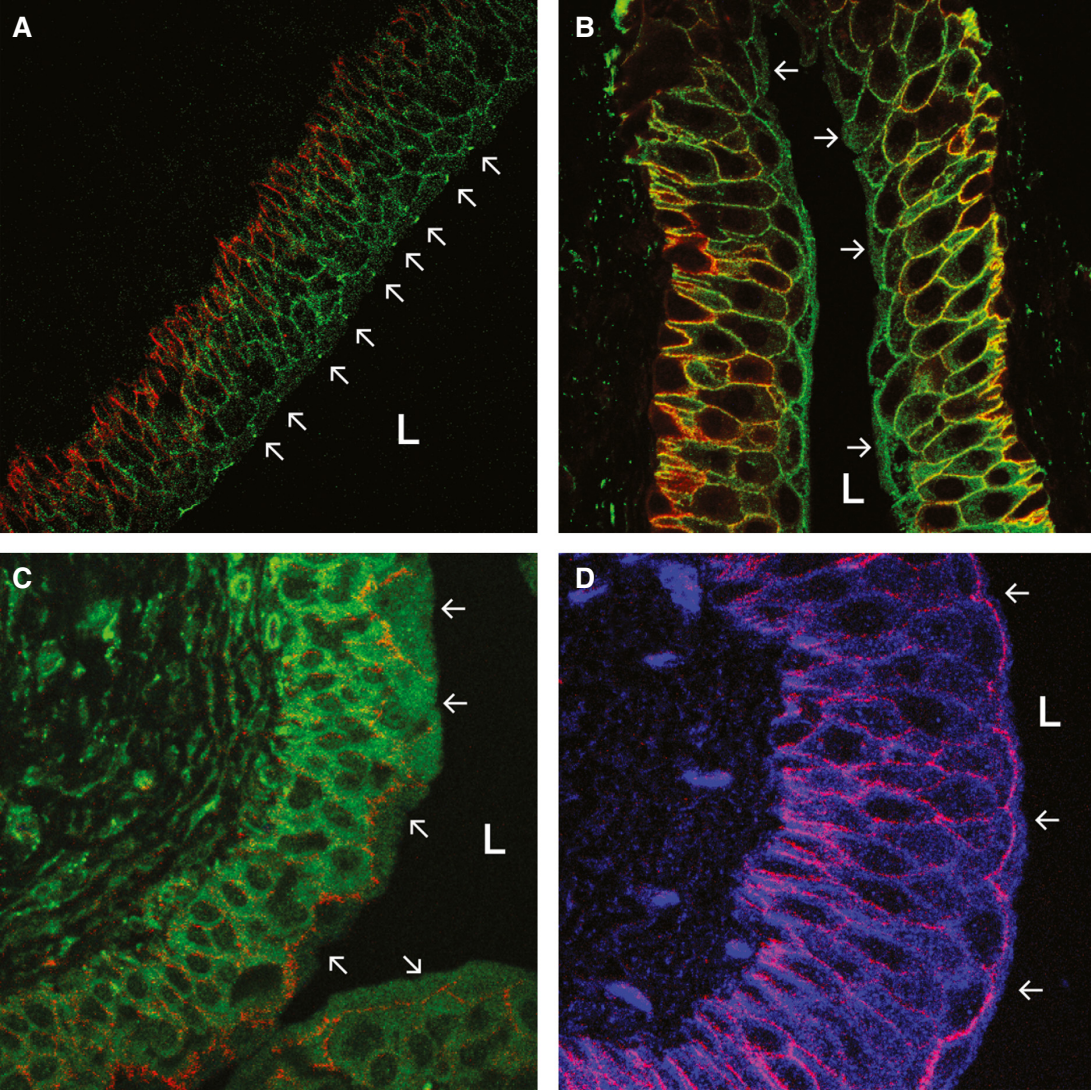
(A) Immunofluorescent confocal microscopy of bear urothelium showing the tight junction protein occludin (green) expressed in tight junctions of adjoining umbrella cells and, to a lesser extent, on all umbrella and intermediate cell basolateral membranes. Arrows indicate tight junctions. Aquaporin-3 (red) stains basal and intermediate cell basolateral membranes, but not umbrella cell apical membrane. L, lumen. (B) Immunofluorescent confocal microscopy of bear urothelium showing UT-B expression (green) on basolateral plasma membranes and to lesser extent in cytoplasm (arrows) of all urothelial cells, but not on umbrella cell apical membrane. Aquaporin -3 =  red. (C) Immunofluorescent confocal microscopy of rat urothelium showing sodium-potassium ATPase expression (in orange-red) on all urothelial cell membranes except umbrella cell apical membrane. ATPase expression is strongest on umbrella cell basolateral membrane. (D) Immunofluorescent confocal microscopy of bear urothelium showing sodium-potassium ATPase expression (in purple-red) on all urothelial cell membranes except umbrella cell apical membrane (arrows). ATPase expression is strongest in umbrella cell basolateral membrane.

Junctional complexes consisting of tight junctions and adherens junctions were identified by electron microscopy at the junction of adjacent umbrella cells apical and lateral membranes in both bears and rats, with desmosomes present deeper in the basolateral membranes (Fig.2A and B). The appearance of the junctional complexes in bears was generally very similar to that in other mammalian species, but in areas in which luminal cells seemed to represent newly exposed and transforming intermediate cells, the junctional complexes sometimes were not apparent, or attenuated (Fig.1E). To determine if bear tight junctions are comprised of the same proteins as described previously in rodent species (Acharya et al. 2004), we carried out immunofluorescent confocal microscopy utilizing representative, well-characterized antibodies to occludin and claudins -2, and -4. By immunofluorescent microscopy, the tight junction protein claudin -2 was not apparently expressed in bear urothelia or (as previously reported in rat, mouse, and rabbit by Acharya et al. (2004)) in rat urothelia (images not shown), although mRNA for claudin-2 was previously identified in mouse urothelia (Acharya et al. 2004). Claudin- 4 was expressed on all bear (red/orange stain Fig.3A; blue stain Fig.3C), and rat (red/orange stain Fig.3E and H) urothelial plasma membranes (excepting the apical membrane), and at their tight junctions as previously described in rats, mice, and rabbits (Acharya et al. 2004). These findings are consistent with prior studies suggesting high resistance and low permeability of urothelial junctions since the presence of claudin -4 is usually associated with low, and claudin -2 with high permeability (Acharya et al. 2004; Angelow et al. 2008). In bears (and rats, not shown), the tight junction protein occludin was expressed in tight junctions and to a lesser extent on all urothelial plasma membranes (green stain Fig.4A) as previously described in rats and mice, but not rabbits, by Acharya and coworkers (Acharya et al. 2004).

### Representative membrane transporters and channels

We carried out immunofluorescent confocal microscopy of bear urothelia to localize expression of several important membrane transporters and channels previously reported in mammalian urothelia. In the bear, as in rats (Spector et al. 2002), aquaporin (AQP) -1 is expressed in suburothelial capillaries and arterioles (data not shown) and AQP -3 expressed in basolateral membranes of all urothelial cells – most strongly in basal and low intermediate cells (red/orange stain Fig.4A and B). As is the case for rats and dogs (Spector et al. 2002, 2004), AQP -3 is not expressed on bear umbrella cell apical membrane. Urea transporter –B (UT-B) in bears is expressed in the urothelial cell cytoplasm and all plasma membranes except the apical membrane (green stain Fig.4B) as previously reported in rats, mice, and dogs (Spector et al. 2004, 2007; Lucien et al. 2005). Sodium potassium ATPase (Na, K, ATPase) was identified by immunofluorescent confocal microscopy in all cell membranes except in the umbrella cell apical membrane of bears (Fig.4D) and is similarly expressed in rats (Fig.4C). In both bear and rats, it appeared that Na, K, ATPase is most strongly expressed in the basolateral membrane of the umbrella cells (Fig.4C and D). Thus, location and intensity of Na, K, ATPase expression in urothelia of bears and rats is similar to that previously reported in humans (Espineda et al. 2003).

## Discussion

This study, the first examining bear urinary bladder, shows that the black bear bladder has a remarkably similar microscopic structure to that of rat and other previously described nonhibernating mammalian species. Thus, the layers comprising bear bladder wall, including the serosal, muscular, subepithelial lamina propria, and in particular the urothelial layer are anatomically similar to other mammalian species. Furthermore, despite areas of cell damage and loss of tissue integrity in bear tissue samples (see below), we were unable to demonstrate substantial light or electron microscopic differences between (nonhibernating) bears and other mammals with regard to the known barriers to potential transurothelial solute and water transport – the umbrella cell (luminal) membrane and the tight junctions between the umbrella cells. We are unable to determine the possible functional significance of our observation that all bears studied had extensive areas of flattened umbrella cells. While this is characteristic of urothelia in distended/stretched bladders in other species, we have no information regarding the state of bladder distension in our bears.

Using confocal immunofluorescent microscopy to localize multiple antibodies to important urothelial membrane and tight junction proteins, we could not identify significant differences in localization or expression between nonhibernating bears and other nonhibernating mammals. Our data are consistent with the conclusion of early workers, based on light, and electron microscopy, that “the permeability barrier based on the luminal membrane is common to the physiologically competent urinary bladder in all mammalian species,” (Firth and Hicks 1973). It must be acknowledged, however, that our choice of antibodies was not exhaustive – and we cannot yet conclude that urothelial differences between bear and nonhibernating mammals do not exist. Further, two critical components of the urothelial permeability barrier(s) also need to be explored: the lipid bilayer component of the umbrella cell apical membrane (Zeidel 1996; Hill and Zeidel 2000, 2003), and the mucin coating overlying the apical membrane – often referred to as the GAGs layer or glycocalyx (Parsons et al. 1990; Hurst 1994; Tajana and Cervigni 2013). Thus, there may be important species-dependent functional and/or compositional variability for any of the components of the permeability barrier. Further, for bears (and possibly other hibernating species), these differences might most likely be altered during the state of hibernation. Interestingly, smaller mammalian hibernators such as ground squirrels undergo frequent brief spontaneous arousals from hibernation during which time they urinate (Jani et al. 2013), and therefore may not need to reabsorb urinary constituents across bladder urothelia.

We also show that urothelial tissues from wild bears euthanized in the field may demonstrate histologic changes characterized by various stages of cell damage and, in some cases, loss of superficial umbrella cells. We think it unlikely that these histologic changes represent the baseline “normal” state in bear urinary bladder (although theoretically an increased propensity to disruption of urothelial integrity and consequently a reduction in barrier functions could account for increased absorption of urinary constituents in both active and hibernating bears). The “very easy loss” of superficial cells from urothelia of postmortem specimens was previously noted in a work devoted to a comparison study of urothelium in a variety of mammals – some wild (Firth and Hicks 1973). While it is possible that some of the histologic changes we describe occurred postmortem as a result of the time between euthanasia in the field and subsequent tissue fixation, it seems likely that at least a portion of the histologic changes may be a reflection of animal stress related to illness, trauma, and/or circumstances surrounding animal capture in all of our animals. Several authors have demonstrated rapid desquamation of urothelial cells following induction of a variety of stress syndromes (Dalal et al. 1994; Jezernik et al. 1995; Veramic and Jezernik 2000; Apodaca et al. 2003). Veramic and coworkers and Dalal et al. noted patchy areas of urothelial desquamation (single or multiple umbrella cells) and dilatation of extra cellular spaces (if urine was in the bladder) following 96 h constant environmental illumination, or single-intraperitoneal injection of stress hormones (norepinephrine and hydrocortisone) in mice (Dalal et al. 1994; Jezernik et al. 1995; Veramic and Jezernik 2000). Apodaca and coworkers demonstrated patchy alterations of umbrella cell cytoplasmic density and other changes, including necrosis and disruption of umbrella cells within 2 h of spinal cord injury (S.C.I.) – followed by regeneration of superficial cells (likely newly exposed intermediate cells) initially having a small cobblestone appearance in rats (Apodaca et al. 2003; Khandelwal et al. 2009). They suggested that the changes in urothelial histology (and, also measured, reduced transepithelial resistance and markedly increased water and urea permeability) were due to the effects of SCI – elicited catecholamines on urothelial tissues (Apodaca et al. 2003). The histologic changes described by these authors are very similar to some of the changes we note in our bear bladder urothelial specimens. Regardless of the cause of the histologic alterations in our bear tissues, however, our data illustrates the difficulties in obtaining pristine urothelial tissues suitable for study from wild animals – particularly from large animals – collected in the field.

We were not able to study urinary tract tissues of euthanized hibernating bears – which are rarely available to state authorities (personal communications Harry Spiker, Black Bear Project Leader, Maryland, to the authors). The one bear (#5) whose tissues were obtained during the winter was sick with mange, had not “fattened” (considered important for successful hibernation) and was active and foraging. Thus, we lack direct data on the urothelial permeability barriers during hibernation. Clearly urinary constituents must pass through one or more of the components of the known permeability barriers. That such transport can occur in mammals which do not undergo hibernation was previously shown (Levinsky and Berliner 1959; Walser et al. 1988) and confirmed by recent studies demonstrating that significant regulated solute and ion transport can occur across (nonhibernating) mammalian urothelia (Spector et al. 2011, 2012, 2013), including humans (Cahill et al. 2003; Shatik et al. 2005). Thus, Spector and coworkers noted urothelial secretion of urinary solutes (sodium, potassium, chloride, urea, creatinine) in water-loaded rats and urothelial reabsorption of these same urinary solutes in both control and (more so in) water-deprived rats (Spector et al. 2011, 2012, 2013). Net transport for solutes was a function of both their urinary concentration and as well a bladder change associated with whole animal hydration status. Animals subjected to water deprivation had significantly greater numerical and percentage reabsorption of all urinary constituents (except water – which had no net transport in spite of an increase in basolateral membrane expression of AQP-3 and AQP-2 (Spector et al. 2002). While simple or facilitated diffusion across the apical membrane likely accounts for at least some of the observed solute transport (since direction and magnitude of transport, in these and in earlier studies, for each solute was similar and dependent on the urine to plasma concentration gradient), the urothelial site and mechanism(s) for the increased solute reabsorption in water-deprived rats remains unclear. Interestingly, in comparing urothelial uroplakins expression between rats and bears, we noticed that “total” and individual uroplakins Ia, IIIa, and IIIb were relatively strongly expressed in bear and in water-loaded rat apical membranes. In rats, this was possibly due to the persistent stretch (imposed on bladder wall during water diuresis), which is known to stimulate exocytosis and apical membrane insertion of cytoplasmic vesicles containing uroplakins (Chang et al. 1994; Truschel et al. 2002; Khandelwal et al. 2009). In contrast, there was strong cytoplasmic expression and little apical expression of uroplakins in water-deprived rats. While this may in part reflect increased apical membrane endocytosis in relatively contracted bladders, it seems possible that a reduction in uroplakins in the apical membrane of dehydrated rats contributes to the significantly increased reabsorption of urinary solutes previously described in water-deprived animals (Spector et al. 2011, 2012, 2013).

Alternatively, it is also possible that endocytic vesicles could be responsible for a trans-apical membrane transport of urinary constituents (Spector et al. 2012). A number of investigators have described luminal fluid markers along with internalized apical membrane components in subapical endosomes and vesicles (Chang et al. 1994; Burton et al. 2002; Grasso and Calderon 2009, 2013b; Khandelwal et al. 2009) and Grasso has described endosomal release of fluid contents into cytosol (Grasso and Calderon 2013a). Given that hypertonicity also stimulates apical membrane endocytosis (Apodaca 2002; Burton et al. 2002), it is interesting that Minsky and Chlapowski reported a “striking positive correlation” in both contracted and dilated bladders between the size and number of subapical cytoplasmic vesicles and the osmolality of urine (Minsky and Chlapowski 1978).

It seems likely that active bear urothelia possess at least the same capabilities for net urothelial transport as other mammalian urothelia and that hibernation might be associated with alteration of components of one or more of the permeability barriers – resulting in an increased capacity for reabsorption – especially for water. Theoretically hibernation might be associated with upregulation of specific umbrella cell membrane channels or transporters (e.g. AQP-3, UT-B, ENaC), new expression of channels and transporters on apical membrane, a reduction in membrane barrier proteins such as uroplakins or claudins, increased intravesical hydrostatic pressures, and/or an increase in endocytic vesicle formation and consequent intracellular release of their urinary contents. Interestingly recent data suggests that dietary factors might potentially affect functions of multiple components of the urothelial permeability barriers, including the apical membrane lipid bilayer and uroplakins (see below), tight junctions (Jiang et al. 1998; Ulluwishewa et al. 2011), membrane solute transporters (Ma and Eaton 2005; Dopico and Bukiya 2014), and even endocytic vessel formation and permeability (Grasso and Calderon 2009, 2013b). For hibernating bears dietary fat has special importance and significance. During the fall season before hibernation, there is a dramatic and critical increase in the quantity of food intake (hyperphagia) and a change to a high fat diet, (Beeman and Pelton 1980; Hellgren et al. 1989), resulting in a 30–35% weight gain due solely to stored fat (Lundberg et al. 1976; Nelson 1978; Barboza et al. 1997; Hellgren 1998; Hissa et al. 1998). During winter hibernation, the stored fat is the exclusive fuel supporting metabolism – such that during hibernation body fat mass falls but lean body mass remains constant (Nelson et al. 1973; Lundberg et al. 1976; Barboza et al. 1997). During black bear hibernation, serum concentrations of cholesterol, phospholipids, triglycerides, and total free fatty acids increase (Nelson 1978, 1980; Ahlquist et al. 1984; Hellgren 1998; Stenvinkel et al. 2013). Interestingly during a similar hibernation in European Brown Bear, the serum concentrations of some individual fatty acids rise, and some fall – the latter including steric, oleic, alpha, and gamma linolenic (“essential” fatty acids) and eicosapentaenoic acid (Hissa et al. 1998). These fatty acids are also a major component of the urothelial apical membrane in rats (Ketterer et al. 1973; Hicks et al. 1974; Calderon and Eynand 2000), but the lipid composition of urothelial membranes in active (and hibernating) bears is unknown.

Importantly in many mammalian species, dietary lipids have been shown to profoundly affect both membrane lipid composition and membrane function (including membrane permeability, activity of membrane – bound enzymes and receptors, and endocytosis and exocytosis (Abeywardena et al. 1984; Awad 1986; Dopico and Bukiya 2014; Spector and Yorek 1985; reviewed in Kummerow 1983 and Grasso and Calderon 2013a), as well as tight junction permeability and occludin expression (Jiang et al. 1998). In urothelial apical membranes in particular, dietary lipids have been shown to alter: membrane fatty acid composition and fluidity (Calderon and Eynand 2000), the membrane leaflet lipid distribution and uroplakin expression and cross-linking (Bongiovanni et al. 2005), the structural array and size of uroplakin proteins comprising the urothelial plaques (Calderon and Grasso 2006) the thickness and ultrastructural asymmetry of the apical and vesicular membranes (Kalinec et al. 1986), the pathways and rates of endocytic recycling from apical membranes (Grasso and Calderon 2013b), and the permeability of endocytic vessels obtained from rat umbrella cell apical membranes (Grasso and Calderon 2009). Finally, investigators have demonstrated in small hibernating mammals that the composition of dietary fats during fall hyperphagia influences the fatty acid composition of tissue depot fats and cellular membranes as well as patterns of hibernation and torpor (Aloia and Raison 1989; Geiser et al. 1994; Dark 2005; Ruf and Arnold 2008). Geiser and others have suggested that the degree of saturation of dietary fatty acids affects the composition of plasma membranes and may influence survival of hibernation (Geiser et al. 1994; Dark 2005). Taken as a whole, the above studies raise the possibility that profound changes in lipid metabolism resulting from both prehibernation hyperphagia and the sole reliance on oxidation of accumulated fat deposits during hibernation may alter the composition and function of bear umbrella cell membrane lipid bilayer, uroplakins, and/or tight junctions – resulting in increased bladder permeability during hibernation.

In summary, during bear hibernation, reabsorption of the daily urine output across the bladder luminal apical membrane would allow metabolic recycling of urinary constituents, including nitrogenous “waste” products and other solutes, water, and ions, thereby obviating the need for eating and drinking. This process is likely critical for successful hibernation in bears. We show that the microscopic anatomy and a representative sample of urothelial barrier proteins, including uroplakins, tight junction proteins, and transporters/channel proteins in active wild bears appear to be remarkably similar to those in nonhibernating mammals. Although obtaining adequate tissue samples from hibernating bears will be challenging, (and may require captive bears) future studies comparing composition and function of urothelial tissues of active and hibernating bears, including measurements of apical membrane lipid and GAG's composition, are warranted to elucidate the mechanism(s) whereby hibernating bears accomplish this remarkable feat.
